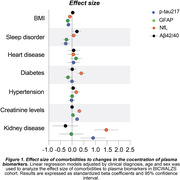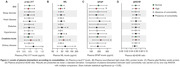# Confounding factors to Alzheimer's disease plasma biomarkers in patients from a memory‐clinic cohort

**DOI:** 10.1002/alz.094091

**Published:** 2025-01-09

**Authors:** Matheus Scarpatto Rodrigues, Markley Oliveira, Firoza Z Lussier, Pamela C.L. Ferreira, Guilherme Bauer‐Negrini, Guilherme Povala, Cynthia Felix, Sarah Abbas, Hussein Zalzale, Carolina Soares, Pampa Saha, Marina Scop Madeiros, Chang Hyung Hong, Hyun Woong Roh, Helmet T. Karim, Jade de Oliveira, Eduardo R. Zimmer, Thomas K Karikari, Bruna Bellaver, Sang Joon Son, Tharick A. Pascoal

**Affiliations:** ^1^ University of Pittsburgh, Pittsburgh, PA USA; ^2^ Department of Psychiatry, University of Pittsburgh School of Medicine, Pittsburgh, PA USA; ^3^ Ajou University School of Medicine, Suwon Korea, Republic of (South); ^4^ Ajou University School of Medicine, Suwon, Gyeonggido Korea, Republic of (South); ^5^ Universidade Federal do Rio Grande do Sul, Porto Alegre Brazil; ^6^ Federal University of Rio Grande do Sul (UFRGS), Porto Alegre, RS Brazil

## Abstract

**Background:**

Blood‐based biomarkers for Alzheimer’s disease (AD) are increasingly prevalent and accessible beyond research environments. Consequently, it is crucial to determine whether confounding factors, particularly highly prevalent comorbidities, influence the levels of these blood biomarkers, thereby refining their clinical interpretation. In this study, we examined the impact of comorbidities on plasma AD biomarker levels within a memory‐clinic cohort.

**Method:**

We analyzed 832 individuals (CU: 101, CI: 731) from the BICWALZS cohort with available information of comorbidities and plasma biomarkers measurements. The effect size of each comorbidity to plasma p‐tau217, neurofilament light chain (NfL) protein, glial fibrillary acidic protein (GFAP) and amyloid‐ß (Aß) 42/40 ratio was accessed by the ß‐estimate and 95% CI values of linear regression models adjusted by clinical diagnosis, age, and sex. Comparison of biomarkers between comorbidities was carried out by ANOVA.

**Result:**

Among the evaluated comorbidities, plasma creatinine levels, BMI index, sleep disorder, and the presence of kidney disease were the primary comorbidities linked to variations in plasma biomarker concentrations (Figure 1). Analyzing the levels of the biomarkers according to the presence of comorbidities, we observed that individuals with increased plasma creatinine exhibited significantly higher plasma levels of p‐tau217, NfL and GFAP in comparison to the individuals with normal levels of creatinine (p < 0.05, Figure 2A, B, C). The mean of p‐tau217 and NfL, but not of GFAP, was elevated between those with kidney disease (p < 0.05, Figure 2A, B). High plasma NfL levels were observed in individuals with diabetes and hypertension (p < 0.05, Figure 2B). On the other hand, high BMI index was associated with lower plasma p‐tau217, NfL and GFAP compared to individual with normal BMI (Figure 2A, B, C). We also observed that individuals with sleep disorder exhibited decreased plasma p‐tau217 levels (Figure 2A). Plasma Aß42/40 ratio was not affected by the analyzed comorbidities (Figure 2D).

**Conclusion:**

Our findings indicate that elevated creatinine levels, a higher BMI index, and the presence of kidney disease are the most significant confounding variables associated with alterations in AD plasma biomarkers. Together, our results shed light on the impact of different comorbidities to the interpretation of biomarker values in real‐world populations.